# α-Cyperone Attenuates H_2_O_2_-Induced Oxidative Stress and Apoptosis in SH-SY5Y Cells *via* Activation of Nrf2

**DOI:** 10.3389/fphar.2020.00281

**Published:** 2020-04-08

**Authors:** Bingxu Huang, Juxiong Liu, Shoupeng Fu, Yufei Zhang, Yuhang Li, Dewei He, Xin Ran, Xuan Yan, Jian Du, Tianyu Meng, Xiyu Gao, Dianfeng Liu

**Affiliations:** ^1^Department of Basic Veterinary Medicine, College of Animal Science and Veterinary Medicine, Jilin University, Changchun, China; ^2^Department of Food Quality and Safety, College of Food Science and Engineering, Jilin University, Changchun, China

**Keywords:** α-cyperone, oxidative stress, apoptosis, ROS, Nrf2

## Abstract

α-Cyperone, extracted from *Cyperus rotundus*, has been reported to inhibit microglia-mediated neuroinflammation. Oxidative stress and apoptosis play crucial roles in the course of Parkinson’s disease (PD). PD is a common neurodegenerative disease characterized by selective death of dopaminergic neurons. This study was designed to investigate the neuroprotective effects of α-cyperone against hydrogen peroxide (H_2_O_2_)-induced oxidative stress and apoptosis in dopaminergic neuronal SH-SY5Y cells. Neurotoxicity was assessed by MTT assay and the measurement of lactic dehydrogenase (LDH) release. The level of reactive oxygen species (ROS) was measured by dichlorodihydrofluorescin diacetate (DCFH-DA) staining. The apoptosis of SH-SY5Y cells was evaluated by annexin-V-FITC staining. The translocation of NF-E2-related factor 2 (Nrf2) was determined by western blot and immunofluorescence staining. Western blot analysis was conducted to determine the expression level of cleaved-caspase-3, the pro-apoptotic factor Bax, and the anti-apoptotic factor, Bcl-2. The results showed that α-cyperone substantially decreased H_2_O_2_-induced death, release of LDH, and the production of ROS in SH-SY5Y cells. In addition, we found that α-cyperone attenuated H_2_O_2_-induced cellular apoptosis. Moreover, α-cyperone remarkably reduced the expression of cleaved-caspase-3 and Bax, and upregulated Bcl-2. Furthermore, α-cyperone enhanced the nuclear translocation of Nrf2. Pretreatment with brusatol (BT, an Nrf2 inhibitor) attenuated α-cyperone-mediated suppression of ROS, cleaved-caspase-3, and Bax, as well as α-cyperone-induced Bcl-2 upregulation in H_2_O_2_-treated SH-SY5Y cells. α-cyperone neuroprotection required Nrf2 activation. In conclusion, α-cyperone attenuated H_2_O_2_-induced oxidative stress and apoptosis in SH-SY5Y cells *via* the activation of Nrf2, suggesting the potential of this compound in the prevention and treatment of PD.

## Introduction

Parkinson’s disease (PD) is the second most common age-associated neurodegenerative disorder, affecting about 2% of subjects older than 60 years and more than 5 million people worldwide (Olanow et al., 2009). PD is mainly characterized by a progressive degeneration of dopaminergic neurons in the substantia nigra pars compacta region of the midbrain (Obeso et al., 2010). Its most common features include resting tremor, rigidity, and bradykinesia (Coelho and Ferreira, 2012). Previous studies have reported that age, genetic, and environmental factors are major determinants in PD pathogenesis. Moreover, the role of oxidative stress and mitochondrial dysfunction is increasingly recognized. For example, it has been demonstrated that oxidative stress and mitochondrial dysfunction can cause neuronal damage and degeneration in PD pathogenesis (Henchcliffe and Beal, 2008; Hauser and Hastings, 2013; Subramaniam and Chesselet, 2013). Therefore, inhibition of these events is a potential strategy to protect dopaminergic neurons.

Oxidative stress is a damaging response resulting from an imbalance between the generation of oxygen-derived radicals and the organism’s antioxidant potential, and implies excessive production of reactive oxygen species (ROS) (Schieber and Chandel, 2014). Excessive ROS production and accumulation damage the structure of cell membranes and the biological functions of lipids, proteins and DNA, and ultimately cause the initiation of cell apoptosis (Ma, 2012). Additionally, hydrogen peroxide (H_2_O_2_)-induced ROS production and release can cause a series of oxidative stress responses, which in turn lead to mitochondrial dysfunction, cell damage, and death (Cheng et al., 2010). Apoptosis induced by mitochondrial dysfunction is regulated by the anti-apoptotic protein, Bcl-2, and the pro-apoptotic protein Bax (D’orsi et al., 2017). Caspase-3, a key component of the apoptotic machinery, is activated to generate cleaved-caspase-3 leading to cell death (Porter and Janicke, 1999). Therefore, suppression of cleaved-caspase-3 and Bax, or Bcl-2 upregulation, may prevent neuronal apoptosis due to oxidative stress.

α-Cyperone, extracted from *Cyperus rotundus*, has been demonstrated to weaken the inflammatory response by microtubule destabilization in the brain (Azimi et al., 2016). Sutalangka et al. has reported *Cyperus rotundus* has neuroprotective and cognitive-enhancing effects in AF64A-treated rats (Sutalangka and Wattanathorn, 2017). We previously showed that α-cyperone exerts neuroprotective effects by inhibiting microglia-mediated neuroinflammation (Huang et al., 2018). The SH-SY5Y neuroblastoma cell line has been previously used as a cellular model of PD (Xie et al., 2010). H_2_O_2_ is commonly used to reproduce oxidative stress *in vitro*. It has been reported that oxidative stress plays a crucial role in PD (Jenner, 2003; Puspita et al., 2017). Some natural products were reported to prevent oxidative stress in H_2_O_2_-treated SH-SY5Y cells (Alvarino et al., 2017; De Oliveira et al., 2017; De Oliveira et al., 2018). However, the effects of α-cyperone in H_2_O_2_-treated SH-SY5Y cells have not been explored. Here, we investigated whether α-cyperone prevented oxidative stress-induced apoptosis of SH-SY5Y cells, and addressed the underlying mechanisms.

## Materials and Methods

### Materials

3-(3,4-Dimethylthiazole-2-yl)-2,5-diphenyltetrazoliumbromide (MTT), brusatol (BT, an Nrf2 inhibitor), and dimethylsulfoxide (DMSO) were obtained from Sigma Aldrich (St Louis, MO, USA). The Bicinchoninic acid protein H_2_O_2_ assay (BCA) kit, RIPA lysis buffer, ROS detection kit, and LDH detection kit were obtained from Beyotime Biotechnology (Shanghai, China). α-Cyperone (> 98% purity; Yuan ye Biotech, Shanghai, China) was dissolved in DMSO and freshly diluted to the final concentration of 0.05% DMSO. The Annexin V-FITC/PI Apoptosis Detection Kit was obtained from Solarbio (Beijing Solarbio Science & Technology, Beijing, China). Phosphate buffered saline (PBS), Fetal bovine serum (FBS) and Dulbecco’s modified Eagle’s medium (DMEM) were obtained from Gibco (Grand Island, NY, United States). Primary antibodies against Nrf2, Bax, Bcl-2, cleaved-caspase-3, PCNA, and β-actin were obtained from Proteintech Group (Wuhan China). Secondary goat anti-rabbit or goat anti-mouse antibodies were obtained from Santa Cruz, CA, USA.

### Cell culture

The SH-SY5Y cells were obtained from the Cell Culture Center at the Institute of Basic Medical Sciences, Chinese Academy of Medical Sciences (Peking, China). The cell lines were seeded on 25 cm^2^ cell culture flasks and maintained in DMEM containing 10% FBS, at 37°C in a 5% CO_2_ incubator. When cells were approximately at 80% confluence, they were subcultured by trypsinization (0.05%, w/v) and seeded into 6-well or 96-well plates. SH-SY5Y cells were cultured for five days with 10 μM retinoic acid (RA) in DMEM plus 1% FBS, 2 mM glutamine and the necessary antibiotics, followed by another five days of culture in serum-free DMEM with BDNF (brain-derived neurotrophic factor, 50 ng/mL), glutamine (2 mM), and antibiotics (P/S). After culture in serum-free DMEM for 4 h, the cells were pretreated with α-cyperone (15 μM or 30 μM) for 2 h and then co-treated with H_2_O_2_ (200 µM) for 24 h. To investigate whether the neuroprotective effect of α-cyperone was associated with the activation of Nrf2, SH-SY5Y cells were pretreated with brusatol (BT, an Nrf2 inhibitor dissolved in 0.05% DMSO) for 4 h, co-treated with α-cyperone (30 μM) for 2 h, and then post-treated with H_2_O_2_ for 24 h. Control cells were cultured in medium containing 0.05% DMSO.

### MTT Assay

SH-SY5Y cells (1 × 10^4^ cells/well) were seeded and cultured into a 96-well plate for 12 h. Cells were supplied with fresh serum-free DMEM for 4 h, pretreated with α-cyperone for 2 h, and then co-treated with H_2_O_2_ for 24 h. The cell viability was determined by MTT assay according to the manufacturer’s instructions. First, MTT (0.5 mg/mL) was transferred to the cell cultures, and the cells were incubated for an additional 4 h. The supernatant was removed, DMSO (150 μL/well) was added, and the plates were shaken for 15 min. The absorbance was measured using an absorbance reader (iMark, BioRad, USA) with a testing wavelength of 570 nm and a reference wavelength of 630 nm.

### LDH Assay

The release of LDH in the medium was determined by an LDH assay kit in accordance with the supplier’s instructions. In brief, α-cyperone was transferred into the medium for 2 h before co-treatment with H_2_O_2_. After stimulation with H_2_O_2_ for 24 h, the culture medium (120 μL/well) was collected and transferred to a new 96-well plate. The reaction solution (60 μL/well) was added, and the plate was shaken for 30 min. The release of LDH was evaluated by measuring the absorbance at 490 nm and 600 nm using an absorbance reader (iMark, BioRad, USA).

### Measurement of Intracellular ROS

Intracellular ROS was measured with a ROS detection kit according to the manufacturer’s instructions. Briefly, SH-SY5Y cells were seeded into 96-well plates (2 × 10^4^ cells/well) for 12 h, and then incubated with serum-free DMEM for 4 h. The cells were pretreated with α-cyperone for 2 h, and then incubated with 50 µM of DCFH-DA for 30 min prior to stimulation with H_2_O_2_ for 10 min to induce ROS generation. The cells were washed twice with PBS (100 μL/well), incubated with PBS (100 μL/well), and then analyzed by a multi-detection reader at excitation and emission wavelengths of 485 and 535 nm, respectively.

### Apoptosis Assay

SH-SY5Y cells apoptosis were assessed by Annexin V-FITC/PI Apoptosis Detection Kit. Briefly, cells (1 × 10^6^ cells/well) were seeded in 6-well plates for 12 h and then pretreated with α-cyperone for 2 h followed by co-treatment with H_2_O_2_ for an additional 24 h. Cells were mildly washed twice with ice-cold PBS, collected, and centrifuged at 1500 rpm for 5 min at 4 °C. Next, cells were harvested, and the percentage of apoptotic cells was assessed by flow cytometry. Gated cells were separated into four quadrants: early apoptotic cells (Annexin positive/PI negative), necrotic cells (Annexin negative/PI positive), late apoptotic cells (Annexin positive/PI positive), and viable cells (Annexin negative/PI negative). The regions LR, UL, UR, and LL represent early apoptotic, necrotic, late apoptotic, and live cells, respectively.

### Western Blot

After treatment with α-cyperone or H_2_O_2_, the cells were harvested and lysed in RIPA lysis buffer containing phenyl-methylsulfonyl fluoride (PMSF). The total protein concentration was evaluated by using a BCA kit with bovine serum albumin as a standard. Equal amounts of proteins (30 µg/well) were separated by a 12% SDS-polyacrylamide gel, and then transferred to polyvinylidene difluoride membranes (PVDF: Millipore, Bedford, MA). The membrane was blocked with 5% (w/v) non-fat milk for 2 h at 25°C. The membrane was incubated with the primary and then with the secondary antibody. The membranes were visualized by the ECL western blot detection system according to the manufacturer’s instruction. The band intensities were quantified using Image J gel analysis software. All experiments were performed in triplicate.

### Immunofluorescence Assay

SH-SY5Y cells were seeded on poly-L-lysine-coated coverslips in 24-well plates for 12 h, and then treated with α-cyperone (30 μM) for 2 h. To analyze the intracellular distribution of Nrf2, the cells were harvested and fixed with Immunol Staining Fix Solution for 10 min. After three washes with PBS, the cells were permeabilized for 10 min, incubated with 5% normal goat serum for 2 h, incubated with the anti-Nrf2 antibody (1:200) overnight, and then washed with PBS. After incubation with the secondary antibody for 1 h, the cells were stained with DAPI. The coverslips were washed with PBS and then mounted onto slides with fluorescent mounting medium. Representative images were chose from 9 fields of view per treatment group.

### Nrf2 Silencing by siRNA Transfection

An Nrf2-specific siRNA was used to knock down Nrf2 expression. SH-SY5Y cells (1 × 105 cells/well) were grown in 6-well plates. After treatment with RA and BDNF, the cells were allowed to reach 50%-60% confluence. For each transfection, 80 pmol of Nrf2 siRNA duplex were diluted into 100 μL of siRNA transfection medium (serum-free Opti-MEM). In a separate tube, 4 μL of the transfection reagent, LipofectamineTM 3000, were diluted into 100 μL of siRNA transfection medium (serum-free Opti-MEM). The dilutions were mixed gently and incubated for 20 min at 25°C. Next, 200 μL of the siRNA/Lipofectamine complex were added to cell culture medium serum-free DMEM (2 mL/well). After siRNA transfection for 12 h, the transfected cells were exposed to α-cyperone. The sequences targeting Nrf2 were as follows: sense, 5′ GAA UUA CAG UGU CUU AAU A 3′; antisense, 5′ UAU UAA GAC ACU GUA AUU C 3′.

### Statistical Analysis

The data were presented as the means ± S.E.M and analyzed with SPSS 19.0 (IBM). The comparisons between the different experimental groups were evaluated by one-way ANOVA, whereas multiple comparisons were performed using the LSD method. *P* values < 0.05 were considered statistically significant.

## Results

### Effect of α-Cyperone on H_2_O_2_-Induced Neurotoxicity in SH-SY5Y Cells

**Figure 1:**
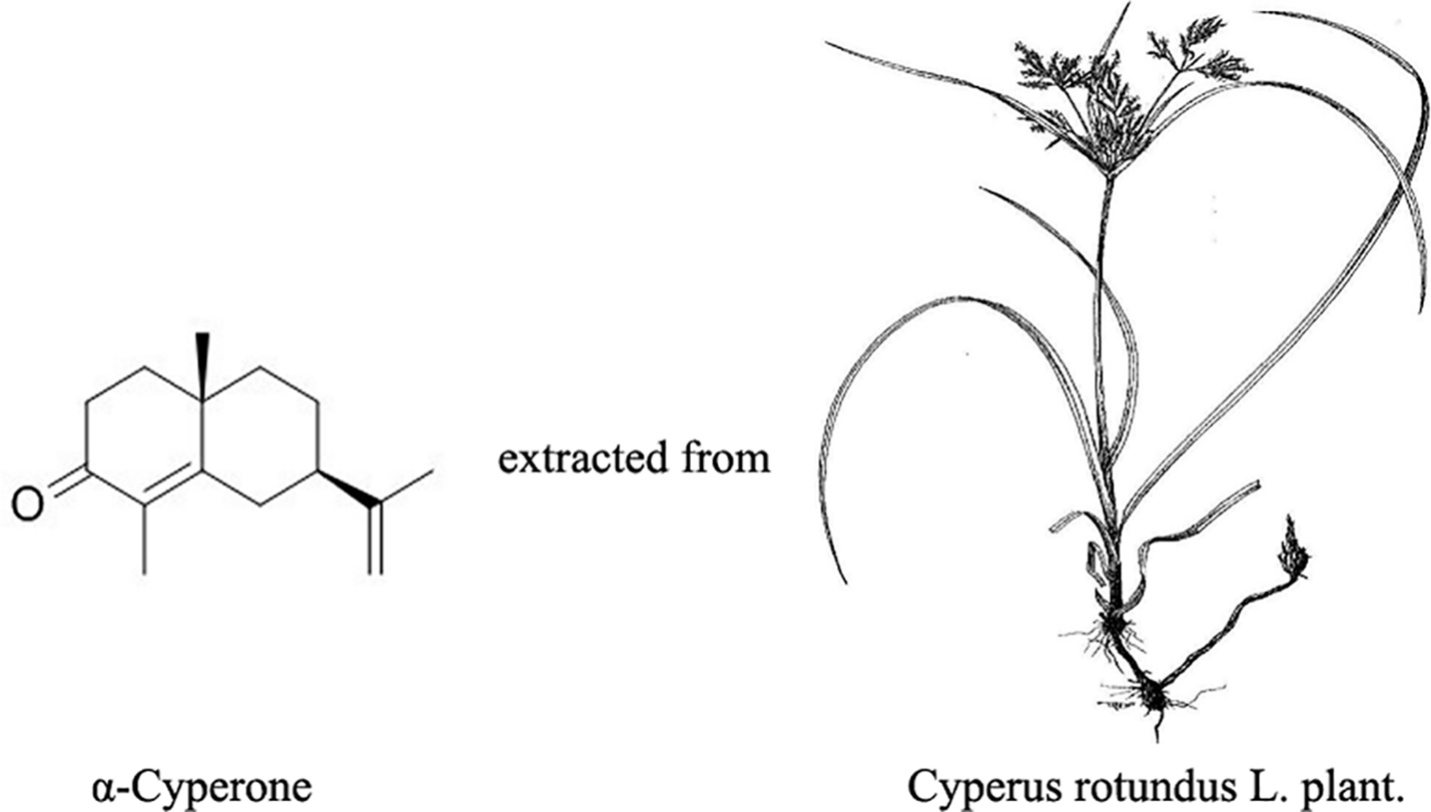
Structure of α-cyperone and *Cyperus rotundus* L. plant. *Cyperus rotundus* L. Traditional uses have been summarized and reported (Pirzada et al., 2015; Kamala et al., 2018).

**Figure 2:**
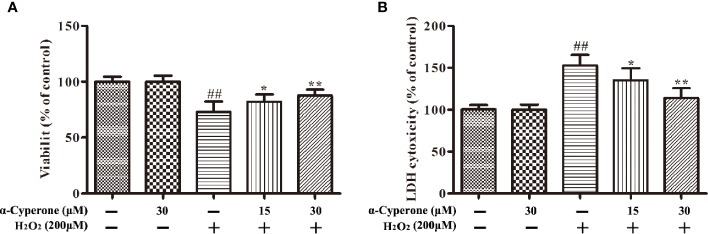
Effect of α-cyperone on H_2_O_2_-induced neurotoxicity in SH-SY5Y cells. After being pretreated with α-cyperone (15 or 30 μM) for 2 h, SH-SY5Y cells were stimulated with H_2_O_2_ (200 μM) for 24 h. **(A)** Cell viability was measured by MTT assay. **(B)** The release of LDH was assessed by LDH assay. All experiments were repeated at least three times and similar results were obtained. Data are presented as the mean ± SE, (n = 5 samples per group). ^##^p < 0.01 vs. the control group, ^*^p < 0.05 and ^**^p < 0.01 vs. the H_2_O_2_-treated group.

To verify possible protective effects of α-cyperone against H_2_O_2_-induced neurotoxicity in SH-SY5Y cells, MTT and LDH assays were performed. In brief, SH-SY5Y cells were pretreated with α-cyperone (15 or 30 μM) for 2 h and then incubated with H_2_O_2_ (200 μM) for 24 h. We found that 30 μM α-cyperone had no toxic effect on SH-SY5Y cells (**Figure 2A**). However, the cell viability was remarkably reduced in H_2_O_2_-treated cells compared to controls (**Figure 2A**). The pretreatment with α-cyperone increased the viability of H_2_O_2_-treated SH-SY5Y cells (**Figure 2A**). The release of LDH, a cytosolic enzyme, is a commonly used indicator of decreased cell integrity. Whereas cell treatment with H_2_O_2_ increased LDH release (**Figure 1B**), pretreatment with α-cyperone markedly attenuated this effect (**Figure 2B**). Taken together, these results revealed that α-cyperone exerted a neuroprotective effect against H_2_O_2_-induced neurotoxicity in SH-SY5Y cells.

### Effect of α-Cyperone on H_2_O_2_-Induced ROS Production in SH-SY5Y Cells

**Figure 3:**
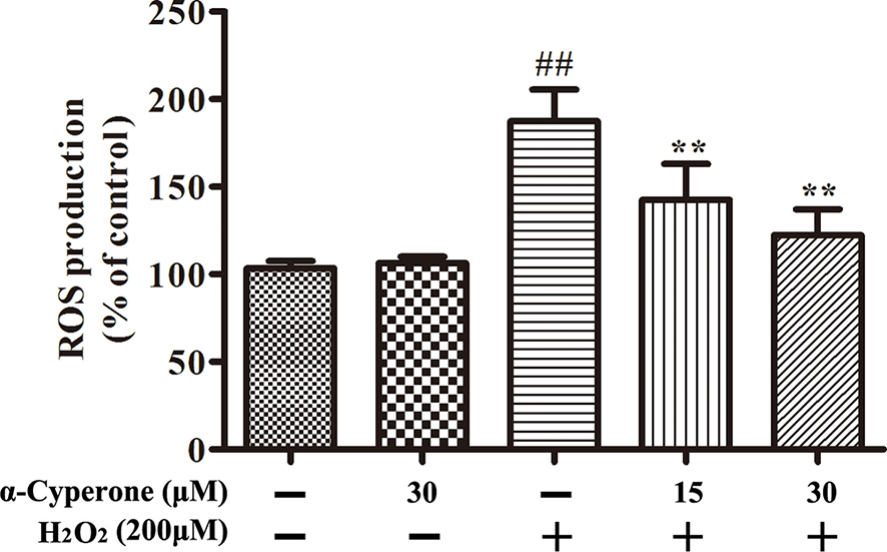
Effect of α-cyperone on H_2_O_2_-induced ROS in SH-SY5Y cells. After treatment with α-cyperone (15 or 30 μM) for 2 h, SH-SY5Y cells were incubated with 50 µM of DCFH-DA for 30 min prior to stimulation with H_2_O_2_ (200 μM) for 10 min. The production of ROS was determined by an ROS detection kit. All experiments were repeated at least three times and similar results were obtained. Data are presented as the mean ± SE, (n= 5 samples per group). ^##^p < 0.01 vs. the control group, ^**^p < 0.01 vs. the H_2_O_2_-treated group.

Mitochondria are the major source of ROS in mammalian cells, and excessive ROS production causes oxidative stress and mitochondrial dysfunction, resulting in cell apoptosis. The production of ROS in SH-SY5Y cells was measured using a DCFH-DA fluorescence assay. We found that 30 μM α-cyperone alone did not affect ROS production (**Figure 3**). Treatment with H_2_O_2_ markedly increased the intracellular level of ROS in SH-SY5Y cells (**Figure 3**). However, pretreatment with α-cyperone dramatically prevented ROS production (**Figure 3**), suggesting that the compound protected dopaminergic neurons against oxidative stress damage.

### Effect of α-Cyperone on H_2_O_2_-Induced Cell Apoptosis

**Figure 4:**
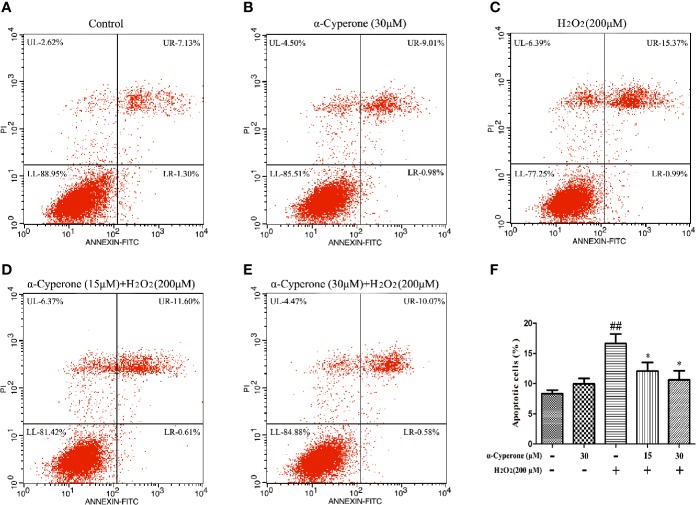
Effect of α-cyperone on H_2_O_2_-induced cell apoptosis. After being pretreated with α-cyperone (15 or 30 μM) for 2 h, SH-SY5Y cells were stimulated with H_2_O_2_ (200 μM) for 24 h. **(A–F)** The apoptosis were assessed by an Annexin V-FITC/PI Apoptosis Detection Kit. All experiments were repeated at least three times and similar results were obtained. Data are presented as the mean ± SE, (n= 5 samples per group). ^##^p < 0.01 vs. the control group, ^*^p < 0.05 vs. the H_2_O_2_-treated group.

To assess the effect of α-cyperone on H_2_O_2_-induced apoptosis in SH-SY5Y cells, the apoptotic rate was measured by flow cytometry. We found that 30 μM α-cyperone alone did not affect the apoptotic rate of SH-SY5Y cells (**Figure 4B**). After treatment with H_2_O_2_ for 24 h, the apoptotic rate of SH-SY5Y cells increased to approximately 50% (**Figure 4C**). However, pretreatment with α-cyperone clearly reduced the H_2_O_2_-induced effect (**Figures 4D–F**), indicating that α-cyperone inhibited H_2_O_2_-induced cell apoptosis. Moreover, whereas the fraction of annexin-negative/PI-positive necrotic cells was about 6.39% after 24 h of incubation with H_2_O_2_, pretreatment with 30 μM α-cyperone brought this proportion to about 4.47%.

### Effect of α-Cyperone on H_2_O_2_-Induced Changes in Bax, Bcl-2, and Cleaved-Caspase-3 Expression

**Figure 5:**
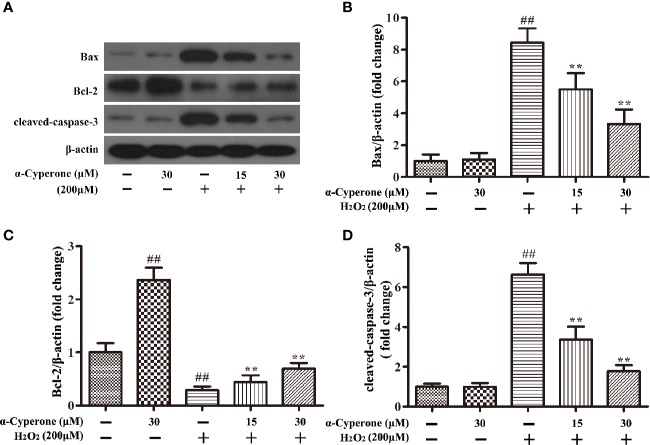
Effect of α-cyperone on Bax, Bcl-2, and cleaved-caspase-3 expression in SH-SY5Y cells pre-challenged with H_2_O_2_. After being pretreated with α-cyperone (15 or 30 μM) for 2 h, SH-SY5Y cells were stimulated with H_2_O_2_ (200 μM) for 24 h. **(A–D)** The expression levels of Bax, Bcl-2, and cleaved-caspase-3 were determined by western blot. All experiments were repeated at least three times and similar results were obtained. Data are presented as the mean ± SE, (n= 5 samples per group). ^##^p < 0.01 vs. the control group, **p < 0.01 vs. the H_2_O_2_-treated group.

To further explore the effects of α-cyperone on H_2_O_2_-induced apoptosis, we analyzed the expression level of Bax, Bcl-2, and cleaved-caspase-3 by western blot. The level of Bax and Bcl-2 expression may be indicative of mitochondrial dysfunction, affecting cell apoptosis. We found that H_2_O_2_ upregulated the pro-apoptotic protein, Bax, while pretreatment with α-cyperone attenuated this effect in SH-SY5Y cells (**Figures 5A, B**). Moreover, 30 μM α-cyperone alone enhanced the expression level of the anti-apoptotic protein, Bcl-2. Notably, while 200 μM H_2_O_2_ downregulated Bcl-2 expression, cell pretreatment with α-cyperone reduced the latter effect (**Figure 5C**). The expression level of cleaved-caspase-3 protein is a marker of cell apoptosis. We found that α-cyperone suppressed cleaved-caspase-3 expression in H_2_O_2_-treated SH-SY5Y cells (**Figure 5D**). Taken together, our results suggested that α-cyperone prevented the apoptosis of dopaminergic neurons due to mitochondrial dysfunction.

### Effect of α-Cyperone on the Nuclear Translocation of Nrf2 in SH-SY5Y Cells

**Figure 6:**
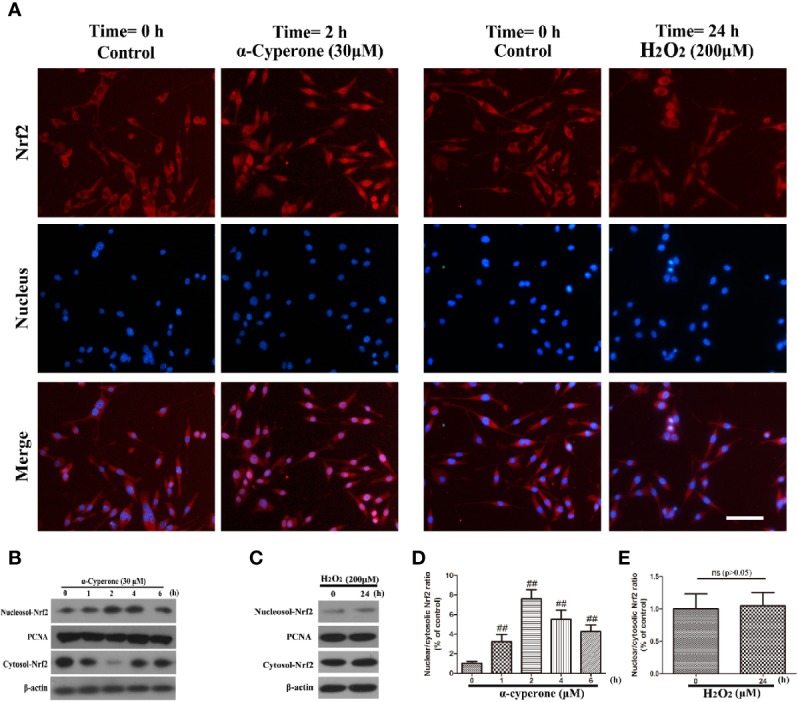
Effects of α-cyperone and H_2_O_2_ on the nuclear translocation of Nrf2 in SH-SY5Y cells. **(A)** SH-SY5Y cells were treated with α-cyperone (30 μM) for 2 h or H_2_O_2_ (200 μM) for 24 h, and the nuclear translocation of Nrf2 was determined by immunofluorescence assay. **(B–E)** After incubation with α-cyperone (30 μM) for various time periods (1-6 h) or with H_2_O_2_ (200 μM) for 24 h, the nuclear and cytosolic Nrf2 levels were measured by western blot. All experiments were repeated at least three times and similar results were obtained. Representative photomicrographs of Nrf2 are shown. The scale bar represents 100 μ. Data are presented as the mean ± SE, (n= 5 samples per group). ^##^p < 0.01 vs. the control group.

Nrf2 is a transcription factor regulating basal and inducible transcription of genes encoding factors protective against various oxidative stresses. Nrf2 activation causes its nuclear translocation, leading to the activation of antioxidant or detoxifying genes. To assess the effect of α-cyperone or H_2_O_2_ on the activation of Nrf2, we used western blot and immunofluorescence assay to measure Nrf2 nuclear translocation in SH-SY5Y cells. We observed that 30 μM α-cyperone enhanced the nuclear translocation of Nrf2 over a period of 6 h (**Figure 6A**). However, the effect of α-cyperone on Nrf2 translocation was already detectable after 2 h. And we also found that 30 μM of α-cyperone observably activated the nuclear translocation of Nrf2 for 2 h (**Figures 6B, D**). In addition, 200 μM H_2_O_2_ had no effect on Nrf2 activation in SH-SY5Y cells, as assessed by both western blot and immunofluorescence (**Figures 6A, C**, **E**). Overall, these results suggested that α-cyperone promoted the nuclear translocation of Nrf2 in SH-SY5Y cells.

### α-Cyperone Effects on ROS Production, as Well as on the Expression of Bax, Cleaved-Caspase-3, and Bcl-2, Depend on Nrf2 Activation

**Figure 7:**
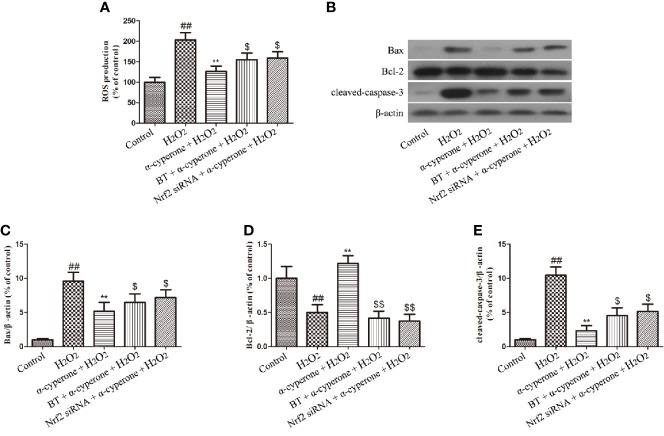
Role of Nrf2 in the protective effects of α-cyperone against H_2_O_2_-induced oxidative stress, mitochondrial dysfunction, and apoptosis. After cell pretreatment with the Nrf2 inhibitor, BT (250 nM), for 4 h or with Nrf2 siRNA for 12 h, SH-SY5Y cells were treated with α-cyperone (30 μM) for 2 h. **(A)** Then, the cells were incubated with 50 µM of DCFH-DA for 30 min prior to stimulation with H_2_O_2_ (200 µM) for 10 min. The production of ROS was determined by ROS detection kits. **(B-E)** The cells were also stimulated with H_2_O_2_ (200 µM) for 24 h, and the expression levels of Bax, Bcl-2, and cleaved-caspase-3 were measured by western blot. All experiments were repeated at least three times and similar results were obtained. Data are presented as the mean ± SE, (n= 5 samples per group). ^##^p < 0.01 vs. the control group, ^**^p < 0.01 vs. the H_2_O_2_-treated group, ^$^p < 0.05 and ^$$^p < 0.01 vs. the α-cyperone + H_2_O_2_ group.

To explore whether α-cyperone effects on oxidative stress, mitochondrial dysfunction, and apoptosis were dependent on Nrf2 activation, we pretreated SH-SY5Y cells with BT (an inhibitor of Nrf2) or knocked down Nrf2 expression by specific siRNAs. Western botting results showed that both conditions dramatically attenuated the inhibitory effect of α-cyperone on ROS production, as well as on Bax and cleaved-caspase-3 expression (**Figures 7A–C**, **E**). Moreover, the inhibition of Nrf2 activation reduced the expression of the anti-apoptotic protein, Bcl-2 (**Figure 7D**). MTT, LDH, and ROS assays revealed the absence of cytotoxicity only in cells cultured in DMEM, control cells, and cells transfected with Nrf2-siRNA (not shown). These results indicated that α-cyperone inhibited oxidative stress, mitochondrial dysfunction, and apoptosis in H_2_O_2_-treated SH-SY5Y cells *via* the activation of Nrf2.

## Discussion

In the present study, we reported the neuroprotective effect of α-cyperone in an *in vitro* model of PD based on the exposure of SH-SY5Y human neuroblastoma cells to H_2_O_2_. This cells have many characteristics of human dopaminergic neuron and, therefore, are commonly employed to *in vitro* mimic PD alterations. Accumulating evidence has demonstrated that oxidative stress, associated with mitochondrial dysfunction, contributes to the development of PD (Dias et al., 2013; Mullin and Schapira, 2013; Yan et al., 2013; Kong et al., 2014). Our results revealed that α-cyperone reduced the excessive production of ROS, and attenuated mitochondrial dysfunction and cellular apoptosis in H_2_O_2_-induced SH-SY5Y cells. Pretreatment with BT (an inhibitor of Nrf2) attenuated the neuroprotective effects of α-cyperone, suggesting that α-cyperone-mediated protection of dopaminergic neurons from oxidative stress-induced apoptosis, as well as from mitochondrial dysfunction, occurred *via* the activation of Nrf2.

We previously reported that α-cyperone inhibits neuroinflammation in activated-microglia. Moreover, it has been reported that α-cyperone inhibits LPS-induced COX-2 expression and PGE2 production through the negative regulation of NF-κB signaling in RAW 264.7 cells (Jung et al., 2013). To further explore whether α-cyperone protected dopaminergic neurons against oxidative stress-induced damage, we investigated the effect of α-cyperone in H_2_O_2_-treated SH-SY5Y cells. We confirmed the neuroprotective effect of α-cyperone against H_2_O_2_-induced neurotoxicity by both MTT and LDH assay. Cell pretreatment with H_2_O_2_ is known to cause the excessive release of ROS, which initiates DNA and mitochondrial damage, resulting in cell apoptosis (Dai et al., 2014; Ding et al., 2016; Fu et al., 2016). Accumulating evidence has shown that ROS production is associated with oxidative stress and mitochondrial anomalies (Nesi et al., 2017; Sundqvist et al., 2017; Franco-Iborra et al., 2018). We found that α-cyperone reduced the production of ROS in H_2_O_2_-treated SH-SY5Y cells. It has been demonstrated that cell apoptosis caused by oxidative stress may be related to various diseases (Cervellati et al., 2014; Paradies et al., 2014; Wu et al., 2014; Li and Hu, 2015). In the present study, α-cyperone notably suppressed the apoptosis of SH-SY5Y cells. To explore this phenomenon, we investigated the effect of α-cyperone on mitochondrial dysfunction. We found that α-cyperone upregulated Bcl-2 and downregulated Bax in H_2_O_2_-treated SH-SY5Y cells, suggesting that α-cyperone suppressed mitochondrial dysfunction. Sanga et al. have proved that the inhibition of cleaved-caspase-3 production protects a SH-SY5Y against toxic injury (Sang et al., 2018). In addition, Wu et al. have reported that carnosic acid protects against 6-hydroxydopamine-induced neurotoxicity in *in vivo* and *in vitro* models of PD, by inducing antioxidative enzymes and inhibiting the production of cleaved-caspase-3 (Wu et al., 2015). We demonstrated that α-cyperone effectively inhibited the expression of cleaved-caspase-3 in H_2_O_2_-induced SH-SY5Y cells. These results suggested that α-cyperone may protect dopaminergic neurons against mitochondrial dysfunction induced by oxidative stress.

The rhizome of *C. rotundus* has been reported to exert anti-apoptotic and anxiolytic activity in SH-SY5Y cells (Awad et al., 2003; Hemanth Kumar et al., 2013). Moreover, Hu et al. have demonstrated that the essential oil from *C. rotundus rhizomes*, containing α-cyperone (38.46%), possesses an excellent antioxidant activity, protects against DNA damage in human neuroblastoma SH-SY5Y cells (Hu et al., 2017). We found that α-cyperone promoted the nuclear translocation of Nrf2 in SH-SY5Y cells. Nrf2 is a potent transcriptional activator, controlling the transcription of many cytoprotective genes in response to oxidative stress (Maruyama et al., 2011; Qaisiya et al., 2014). Under normal conditions, Nrf2 is present in the cell cytoplasm and maintains the basal levels of cytoprotective enzymes. Upon activation, Nrf2 translocates to the nucleus and upregulates various cytoprotective enzymes (Baird and Dinkova-Kostova, 2011). Several studies have reported Nrf2-dependent neuroprotective effects of natural products in SH-SY5Y cells (Lou et al., 2014; Jin et al., 2015; Kwon et al., 2015; De Oliveira et al., 2016). We found that cell pretreatment with BT (an inhibitor of Nrf2) attenuated α-cyperone-induced neuroprotection.

ROS may be a critical mediator of H_2_O_2_ neurotoxicity. Mitochondria, endoplasmic reticulum, α-synuclein, and dopamine may contribute to oxidative stress in dopaminergic neurons. Our results showed that BT or Nrf2 silencing attenuated α-cyperone effects on cleaved caspase-3 expression in H_2_O_2_-treated SH-SY5Y cells (**Figure 7E**). However, α-cyperone neuroprotection may be associated with other signaling pathways and need be further explored. PD models based on Nrf2 knockout mouse will be a suitable tool to in-depth characterize α-cyperone neuroprotection and its therapeutic potential in the context of PD.

**Figure 8:**
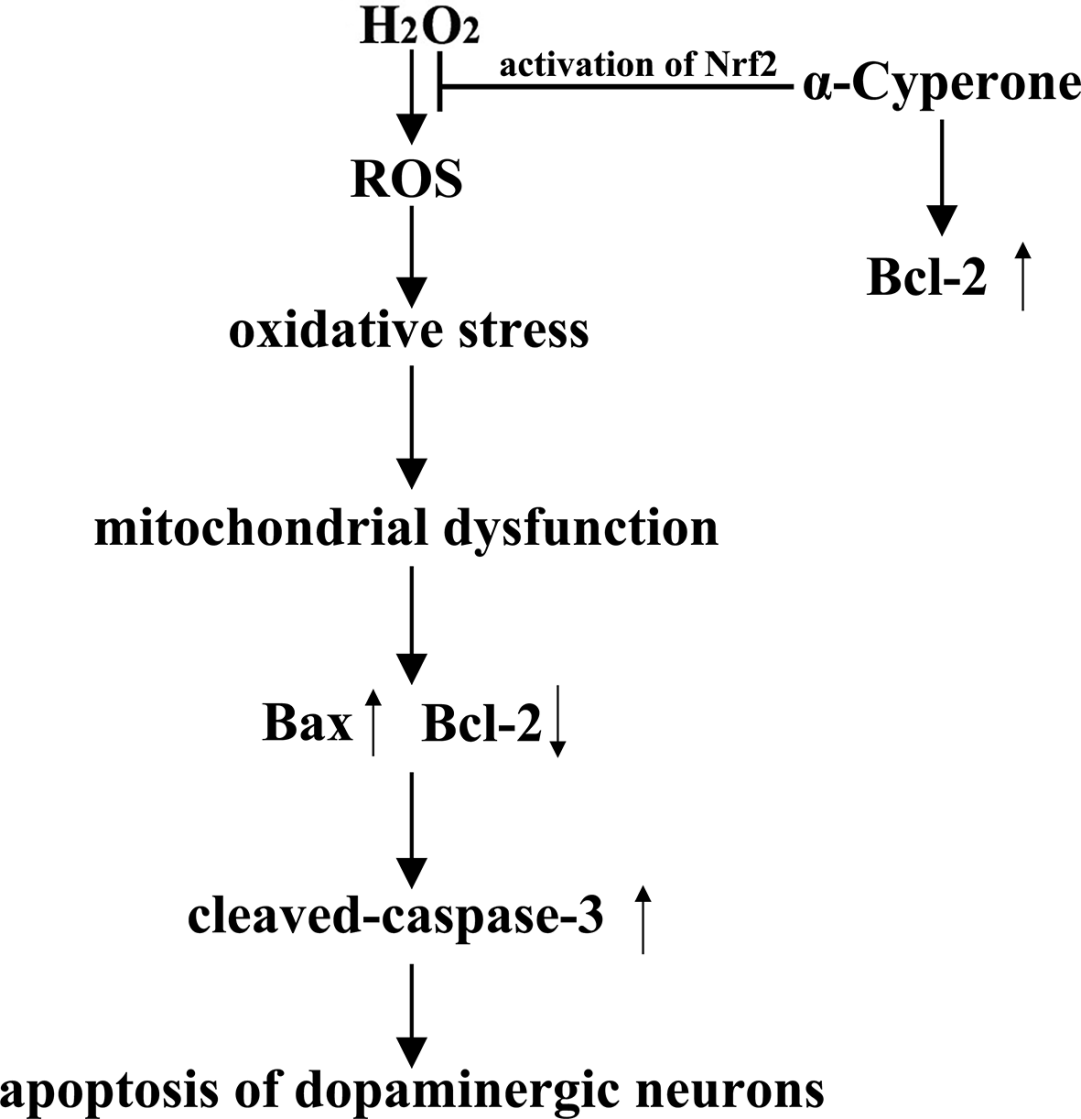
Proposed schematic mechanisms of α-cyperone neuroprotection in an *in vitro* PD model. α-cyperone attenuates H_2_O_2_-induced oxidative stress and apoptosis in SH-SY5Y cells *via* the activation of Nrf2.

In conclusion, our results showed that α-cyperone exerted neuroprotective effects against H_2_O_2_-induced cell apoptosis in SH-SY5Y cells. In addition, α-cyperone inhibited excessive ROS production and mitochondrial dysfunction. Moreover, α-cyperone downregulated the expression of Bax and cleaved-caspase-3, and upregulated Bcl-2 expression. Finally, Nrf2 inhibition attenuated α-cyperone-induced neuroprotection. The neuroprotective actions of α-cyperone are summarized in **Figure 8**. Taken together, these results suggest that α-cyperone is a potential candidate for the prevention of apoptosis in dopaminergic neurons.

## Data Availability Statement

The datasets generated for this study are available on request to the corresponding author.

## Author Contributions

JL and DL conceived and designed the study. BH and SF performed the experiments, analyzed the data, and wrote the paper. YL, DH, XR, XY, TM, JD, XG, and YZ collected the samples and information. All authors reviewed the manuscript. In addition, all authors have read and approved the manuscript.

## Funding

This work was funded by National Nature Science Foundation of China (project No. 31772547, 31672509), Jilin Scientific and Technological Development Program (project No. 20170623029 TC, 20170623083-04TC), JLU Science and Technology Innovative Research Team.

## Conflict of Interest

The authors declare that the research was conducted in the absence of any commercial or financial relationships that could be construed as a potential conflict of interest.
